# An Active-Inference Approach to Second-Person Neuroscience

**DOI:** 10.1177/17456916231188000

**Published:** 2023-08-11

**Authors:** Konrad Lehmann, Dimitris Bolis, Karl J. Friston, Leonhard Schilbach, Maxwell J. D. Ramstead, Philipp Kanske

**Affiliations:** 1 Clinical Psychology and Behavioral Neuroscience, Faculty of Psychology, Technische Universität Dresden, Germany; 2 Laboratory for Autism and Neurodevelopmental Disorders, Center for Neuroscience and Cognitive Systems @UniTn, Istituto Italiano di Tecnologia, Rovereto, Italy; 3 Independent Max Planck Research Group for Social Neuroscience, Max Planck Institute of Psychiatry, Munich, Germany; 4 National Institute for Physiological Sciences, Okazaki, Japan; 5 Centre for Philosophy of Science, University of Lisbon, Portugal; 6 Wellcome Centre for Human Neuroimaging, University College London, UK; 7 VERSES AI Research Lab, Los Angeles, CA, USA; 8 Department of Psychiatry and Psychotherapy, University Hospital, Ludwig Maximilians Universität, Munich, Germany; 9 Department of General Psychiatry 2, Clinics of the Heinrich Heine University Düsseldorf, Germany

**Keywords:** social interaction, second-person neuroscience, active inference, mentalizing, theory of mind

## Abstract

Social neuroscience has often been criticized for approaching the investigation of the neural processes that enable social interaction and cognition from a passive, detached, third-person perspective, without involving any real-time social interaction. With the emergence of *second-person neuroscience*, investigators have uncovered the unique complexity of neural-activation patterns in actual, real-time interaction. Social cognition that occurs during social interaction is fundamentally different from that unfolding during social observation. However, it remains unclear how the neural correlates of social interaction are to be interpreted. Here, we leverage the active-inference framework to shed light on the mechanisms at play during social interaction in second-person neuroscience studies. Specifically, we show how counterfactually rich mutual predictions, real-time bodily adaptation, and policy selection explain activation in components of the default mode, salience, and frontoparietal networks of the brain, as well as in the basal ganglia. We further argue that these processes constitute the crucial neural processes that underwrite bona fide social interaction. By placing the experimental approach of second-person neuroscience on the theoretical foundation of the active-inference framework, we inform the field of social neuroscience about the mechanisms of real-life interactions. We thereby contribute to the theoretical foundations of empirical second-person neuroscience.

Humans are endowed with an intrinsic and irresistible drive to seek social contact and companionship, leading them to engage in (sometimes quite complex) interactions and relationships with their conspecifics (Tomasello et al., 2005). Early on, people are shaped by their caretakers’ and teachers’ guidance and by their experiences of play and exchange with peers, from which they learn how to connect and get along with each other (Wertsch, 1979). Unsurprisingly, separation from our social environment affects our well-being detrimentally, increases the risk of suffering mental- and physical-health issues significantly, and even raises rates of mortality (Cacioppo et al., 2010; Fowler et al., 2013; Hawkley & Cacioppo, 2010; Holt-Lunstad et al., 2015).

A large body of research aims to advance our understanding of those neural processes and mechanisms that enable social interaction and cognition, albeit with varying degrees of genuine interaction between participants (Hari et al., 2015). The increasingly popular scientific approach of *second-person neuroscience* aims to study social cognition in a manner that focuses on human social interaction (Becchio et al., 2010; Di Paolo & De Jaegher, 2012; Hari et al., 2015; Hari & Kujala, 2009; Konvalinka & Roepstorff, 2012; Lehmann et al., 2019; Redcay & Schilbach, 2019; Schilbach, 2010; Schilbach et al., 2013). Second-person neuroscience aims to capture the dynamics and mechanisms at play in genuinely interactive human behavior in individuals and groups. To this end, this paradigm deploys experimental setups that provide participants with opportunities to engage in reciprocal, ideally real-time, social interaction (see Fig. 1). This turn—to the study of the neural mechanisms of social interaction—may seem like an obvious one, but it has not been undertaken until recently: In fact, because of various technological but also conceptual limitations, second-person interactive methodology remains the exception rather than the rule in neuroscience (cf. Bolis & Schilbach, 2020a, 2020b; Schilbach et al., 2013).

**Fig. 1.**
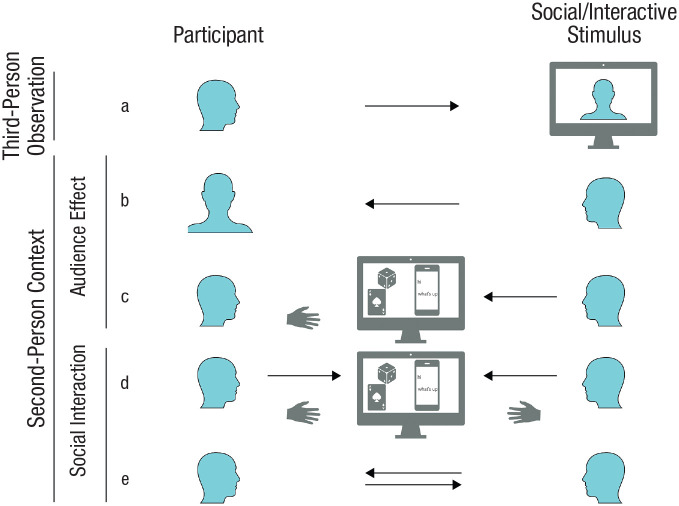
Exemplary setups for social-neuroscience studies. In (a), a classic approach is shown employing third-person observation of a social stimulus; in (b) through (e), a second-person context is shown in which another person is aware of the participant. In (b) and (c), audience-effect setups are displayed in which a participant becomes the object of another person’s attention: (b) shows mere observation of the participant, whereas (c) shows observation of the participant’s actions, usually visible on a computer screen. In (d) through (e), setups for reciprocal social interaction are displayed, with (d) showing slowly paced sequential interactions, usually mediated by a virtual interactional space, and with (e) showing reciprocal and dynamic social interaction.

Several studies have reported differences in behavioral and neural responses between socially interactive (second-person) and observational (third-person) contexts (for reviews, see Feng et al., 2021; Gomez-Marin & Ghazanfar, 2019; Hari et al., 2015; Lehmann et al., 2019; Redcay & Schilbach, 2019; Risko et al., 2012, 2016). To make this more tangible, we discuss two examples here. On a behavioral level, Laidlaw and colleagues (2011) demonstrated that social attention, as indexed by gaze behavior, is significantly decreased when a stranger is actually sitting in a chair across from the participant, compared with when that stranger is merely being displayed on a computer screen and is observed passively. In fact, participants showed more inclination to direct their gaze in the direction of an empty chair than when it was occupied by a person. Another exemplary study by Redcay and colleagues (2010) examined the impact of interaction at a neural level by measuring gaze-based interaction between two people, as compared with viewing the same visual information without the other participant being able to respond. In the interactive condition, the neural-activation pattern showed a widespread increase in activity in the medial prefrontal cortex (mPFC) and the temporoparietal junction (TPJ; as part of the default mode network); the anterior cingulate cortex (ACC) and the anterior insula (AI; as part of the salience network); and subcortical areas, including ventral parts of the basal ganglia. Across studies, these regions have consistently been reported to be activated in interactive versus observational contexts (Cavallo et al., 2015; Rauchbauer et al., 2019; Xie et al., 2020). A recent large-scale meta-analysis comparing social to nonsocial interactions corroborated these findings (Feng et al., 2021).

Despite the reported fundamental differences between live reciprocal interaction and third-person observation, the mechanisms driving these differences are not well understood. This is due to the fact that true social interaction is hard to control experimentally because it is a complex dynamical system that is self-organized and, therefore, takes on a life of its own (De Jaegher et al., 2010; Froese & Gallagher, 2012). As a result, researchers sometimes face difficulties in the interpretation of certain activation patterns. For example, activation in the mentalizing network in second-person neuroscience studies often cannot be clearly interpreted, as the putative underlying processes had not been targeted explicitly (for similar arguments, see Alkire et al., 2018; Redcay & Schilbach, 2019). This speaks for a lack of theoretical and methodological principles to help explain the cognitive and neural mechanisms of social cognition in social interaction. Computational approaches could resolve some of these problems and help to model interaction dynamics and extract precise predictions, which could then be brought to experimental testing. Consequently, the turn to second-person methods aligns with a recent call for “greater theoretical attention to how the emergent properties of an interaction would be reflected in neural activity” (Redcay & Schilbach, 2019, p. 503). The interactive approach of second-person neuroscience also helps to address the critiques—mainly stemming from pragmatist, embodied, and enactive approaches to cognitive science—that have encouraged practitioners in the field to focus on the neural and cognitive mechanisms of real-time social interactions, thereby going beyond the mere observation of social stimuli (Engel et al., 2013; Gallagher, 2005; Newen et al., 2018; Thompson, 2010; Thompson & Varela, 2001).

The aim of this article is to assemble the theoretical (i.e., computational) toolboxes of second-person neuroscience. By doing so, we address the question of what the constituents of genuine social interaction really are. This could inform the debate in social neuroscience about the social specificity of processes involved in social interaction (e.g., Lockwood et al., 2020; Suthaharan & Corlett, 2023). To do this, we leverage the active-inference framework (Friston et al., 2011), which is an increasingly popular approach to computational modeling of complex systems, based on a variational principle called the *free-energy principle* (Ramstead et al., 2018, 2023). The active-inference framework provides tools that allow us to model how embodied agents interpret and proactively interact with salient features of their environment (Bruineberg, Rietveld, et al., 2018; Friston, 2012, 2013a), which, in the case of humans, crucially includes other human agents (Bolis & Schilbach, 2020a; Veissière et al., 2020). Active-inference models formalize the dynamics of the brain, claiming that it instantiates a generative model: The networks of the brain, on this view, produce predictions of what should be sensed next. These predictions are compared against sensory input and (subpersonal Bayesian) beliefs—on which predictions are based—are updated when error or discrepancy is detected. This results in Bayes-optimal inference about the most likely cause of the sensory input (Ramstead et al., 2019). Crucially, the causes of sensory input can include the agent’s own action, which means actions or plans are also inferred, leading to active (planning as) inference (Attias, 2003; Botvinick & Toussaint, 2012; Da Costa et al., 2020). The ideas behind second-person neuroscience and active inference fit together naturally: Both promote the idea that cognition always involves a kind of proactive engagement and prediction of the environment, rather than passive consumption of external stimuli (Bolis & Schilbach, 2020a; Friston et al., 2016; Schilbach et al., 2013).

The remainder of the paper goes as follows. First, we briefly introduce second-person approaches in social neuroscience and the active-inference framework. Second, we examine the idea that a participant’s ability to act toward an interactive partner results in recursive and counterfactual decision-making (i.e., a kind of deep tree search) about possible, situationally appropriate actions. We review literature that examines how this generates patterns of neural activity that one observes in second-person neuroscience studies. We then examine how these action possibilities recruit brain regions that are thought to be responsible for the regulation of the interoceptive and proprioceptive bodily milieu. In the last section, we provide an integrative overview of our argument and propose that the active-inference framework provides a novel perspective on studying second-person neuroscience.

## Second-Person Approaches in Social Neuroscience

The central praxis of second-person approaches in social neuroscience is to remove the barrier between the participant and the social stimuli in such a way that the participant’s role shifts from being a passive observer to becoming an active social agent that can be addressed by another participant in real time (Reddy, 2003; Risko et al., 2016; Schilbach et al., 2013). This entails that information can flow from one participant to another and back. Linguistically speaking, in such a social context, another person can be addressed as “you” (second-person perspective) rather than referring to this person as “she/he/they” (third-person perspective) (Reddy & Morris, 2004); additionally, the subjective first person “I” is also extended to an objective “me” (indicating that “I” has become the object of attention) and a plural “we.” Classic approaches employing third-person observation typically present static or dynamic displays of social stimuli (e.g., pictures or videos of faces or social scenes; Fig. 1a). By contrast, in a second-person context (Figs. 1b–e), participants are embedded in a social situation with another person, who is aware of the participant.

Different manifestations of the second-person approach in social-neuroscience studies vary strongly in the degree to which they resemble real-life social interaction in terms of reciprocity and temporal dynamics (Hari et al., 2015; Redcay & Schilbach, 2019; Shamay-Tsoory & Mendelsohn, 2019). One line of research has studied audience effects, investigating how people behave when they are an object of another person’s attention (Figs. 1b and 1c; Hamilton & Lind, 2016). This may be considered a precursor to reciprocal social interaction and can be subdivided into contexts in which (a) a participant is merely observed (Fig. 1b; e.g., via camera; Somerville et al., 2013) and (b) actions of a participant are observed (Fig. 1c). In the latter case, typically the results of those actions are visible and mediated via a virtual space (e.g., the performance in an estimation task; Müller-Pinzler et al., 2015). Reciprocal social interactions (Figs. 1d and 1e) can vary in the degree to which information flow is dynamic. On one end, there are rather slowly paced tasks, in which participants act sequentially; the task may involve playing a turn-based, dyadic game (e.g., the prisoner’s dilemma; Engemann et al., 2012; Krach et al., 2009) or exchanging chat messages (e.g., Warnell et al., 2017), both of which are mediated by a virtual interactional space (Fig. 1d). On the other end, reciprocal and dynamic social interaction allows for simultaneous behavior and is typically realized via a live video feed (Fig. 1e; e.g., Redcay et al., 2010).

## Active Inference

### The basic idea

In the past decades, there has been a shift away from conceptions of the brain as a passive organ that merely awaits and reactively processes bottom-up sensory input to conceptions that emphasize the fact that cognition and perception find themselves in a mutual embrace with action. These are enactive, ecological, pragmatist, and embodied approaches to the study of the brain (Bolis & Schilbach, 2020a; Bruineberg, Kiverstein, & Rietveld, 2018; Engel et al., 2015; Ramstead et al., 2019; Thompson, 2010). One version of this view casts the brain as an organ that actively generates predictions of its environment (Friston, 2013b). In this model, the dynamics of the brain are said to embody or instantiate a generative model that generates predictions that are compared against sensory input, resulting in a Bayes-optimal inference about the most likely cause of the sensory input (Ramstead et al., 2019). According to the active-inference framework, these predictions are compared with sensory input continuously throughout the hierarchical networks of the brain (Friston, 2005, 2008; Shipp et al., 2013). To model the extrapersonal and internal (i.e., bodily) world in an optimal way, the brain is thought to minimize prediction error throughout the hierarchical generative model.

Heuristically, in this view, when predictions and sensory data clash, conflict is resolved in one of two ways—either by updating one’s beliefs in a Bayes-optimal manner (perception and learning; i.e., changing the internal model to make it more predictive of current sensory input) or by changing the world to make future data consistent with one’s expectations (i.e., action; Friston et al., 2010, 2015). In this view, bodily movements (and autonomic responses) are mediated by lower-level reflexes that fulfil higher-level predictions, forecasting how the proprioceptive (and interoceptive) sensorium ought to feel (Adams et al., 2013; Seth & Friston, 2016). This enactive kind of predictive processing (Clark, 2015) is thought to take place unconsciously at all levels of brain activity, regulating the entire body in its interaction with the environment and allowing it to remain within viable states—those congruent with its survival and thriving (Ainley et al., 2016; Allen et al., 2022; Barrett, 2017; Barrett & Simmons, 2015; Hohwy & Michael, 2017).

In this view, the brain is perpetually engaged in a recurrent message passing between lower and higher levels of the cortical hierarchy. Here, lower levels operate at smaller spatial and shorter temporal scales (e.g., the velocity of people’s movements, or their finer facial features during speech), whereas higher levels or deeper layers process increasingly abstract information at greater spatiotemporal scales (e.g., a person’s temperament and relatively more stable personality traits, or the general layout of facial morphology). Accordingly, predictions at a higher level of the hierarchy suppress or resolve prediction errors in lower levels, as one can observe in the phenomenon of *repetition suppression* (Murray et al., 2002; Summerfield et al., 2008). The balancing act between sensory and prior prediction errors is assumed to be mediated by neuromodulatory mechanisms of synaptic gain that encode their reliability or precision (H. Feldman & Friston, 2010; Moran et al., 2013; Parr & Friston, 2017). This is known as *precision weighting*. According to one version of this theory, called *hierarchical predictive coding*, deeper layers of the cortical hierarchy issue predictions—thought to originate from deep pyramidal cells—about expectations at a lower level of the hierarchy. The resulting prediction errors are, in turn, propagated—by superficial pyramidal cells—to higher levels, updating expectations and ensuing predictions in order to optimize the representation of the hidden states of the world and body (Bastos et al., 2012; Friston & Kiebel, 2009; Shipp, 2016).

### Generative models and active inference

**Fig. 2.**
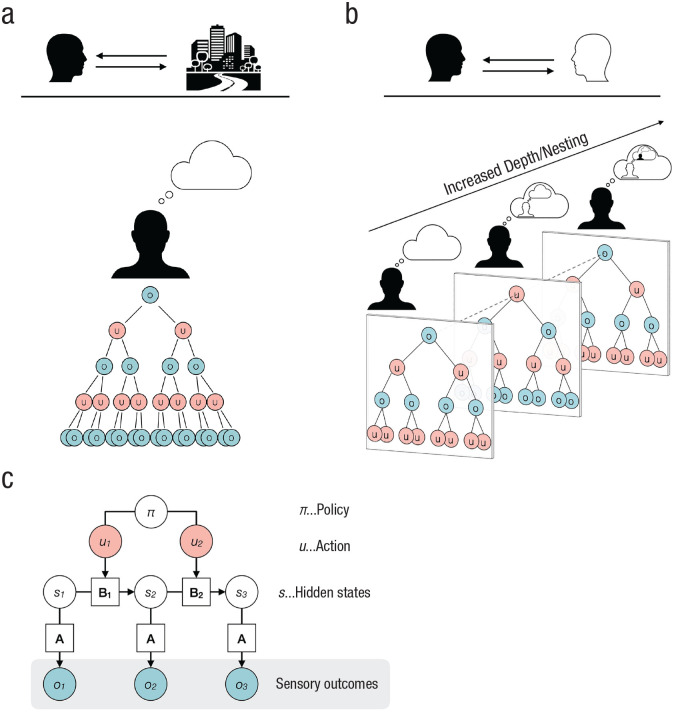
Deep tree exploration of possible policies. The deep tree represents allowable actions (*u*, depicted as red circles) at various time points and their expected sensory outcomes (*o*, depicted as blue circles). In (a) we show deep tree exploration of policies by an agent interacting with a monadic environment; in (b) we show deep tree exploration of policies by an agent interacting with another agent (i.e., dyadic environment). The recursive nature of policy search additionally becomes nested. Own actions constitute observations by another agent (second column), and knowledge about this dependency informs policy selection on a higher level. By coupling agents, policy search and selection becomes increasingly complex due to an increased depth. In the generative model (c), observations (*o*, or sensory outcomes) are caused by hidden states (*s*) that are not observable. The relation between the sensory outcomes and the hidden states is expressed by the likelihood mapping **A** that informs about the likelihood of an observation given a certain state. The hidden states can evolve over time (e.g., from State 1 to State 2). This transition between states (or rather the beliefs about state transitions) is captured by **B**. The respective transitions can be controlled via action (*u*). A series of such state transitions is called a policy (denoted as π).

In this section, we briefly describe the model that generates predicted observations of the (social) environment. We want to provide an intuitive account for a broad audience but refer the interested reader to publications that have elaborated more formal descriptions (e.g., Friston et al., 2021; Hesp et al., 2021; Sandved-Smith, 2021). In Figure 2c, we show a graphical model of a Markov decision process that is commonly used as a generative model for Bayesian inference over discrete states.

In this model, observations (*o* in Fig. 2c, or sensory outcomes) are caused by hidden states (*s*) that are not observable and need to be inferred. The relation between the sensory outcomes and the hidden states is expressed by a likelihood mapping **A** (which is technically a matrix) that encodes the likelihood of an observation, given a certain state or cause (e.g., how likely it is that the sigh of a colleague is indicative of stress). The hidden states, on their part, possess temporal dynamics and evolve over time (e.g., from State 1 to State 2). This transition between states, or rather the beliefs about state transitions, is captured by **B** (also technically a matrix). Interestingly, as states can evolve from one state to another, the respective transitions depend upon action, which has to be inferred. More precisely, an agent can only infer her actions based upon observable consequences. However, she can also have precise prior beliefs about the kinds of actions she would engage in—and thereby gain control over exchange with the environment. A series of controllable state transitions is called a *policy* (denoted as π). Prior beliefs that lead to policy selection are based upon the surprise or free energy expected under a particular policy. Effectively, this means that agents operating under this kind of generative model will act to resolve uncertainty and avoid surprising outcomes (e.g., pain, embarrassment, disorientation).

## Active Inference and Social Action

The most important way of reducing prediction error is to act upon the environment to change the states we want to infer. This is captured very nicely in idiomatic expressions such as the German “*Probieren geht über Studieren*” (literally, “trying is above studying”) or its English equivalent, “the proof of the pudding is in the eating.” To optimally reduce uncertainty via action, one has to select the best option among a variety of possible actions. The obvious problem is figuring out which action will reduce an agent’s uncertainty the most.

### The exploration of opportunities for action

In active inference, this problem is often solved through *counterfactual processing* (Corcoran et al., 2020; Friston, 2018; Palmer et al., 2015)—that is, imagining what the next sensory input would be if I were to execute a particular action. Given some prior beliefs about the typical sensory consequences of actions, the selection among a variety of actions becomes tractable, as an agent must only infer how best to get to preferred and informative sensory data and then pursue the policy that leads to those preferred sensory states. Going a step further, action selection can become “sophisticated,” in the sense that one does not only select actions based on beliefs of the sensory consequences but also based on the consequences of an action for future beliefs (Friston et al., 2021; Hesp et al., 2020). The sophistication lies in having beliefs about beliefs (Costa-Gomes et al., 2001). Sophisticated inference is necessary for resolving epistemic uncertainty, that is, uncertainty caused by a lack of knowledge, requiring additional information to get to a precise belief about the relevant state of the world (Hüllermeier & Waegeman, 2021). A simple example would be the following. You want to bake a cake for a celebration at work, but you are unsure whether you have eggs in the fridge. Checking whether there are eggs in the fridge (disambiguating action 1) informs you about the current environmental state and impacts your subsequent beliefs—that is, whether you want to bake a lemon poppy loaf (sensory consequences of action 2a) if you do have eggs or want to make a cheesecake (sensory consequences of action 2b) if you do not have eggs.

Potential action trajectories are recursively explored, and their sensory consequences of hidden states are counterfactually imagined. This process can span over timescales and has close connections to episodic prospection, rumination, and mind wandering (Christoff et al., 2016; Nolen-Hoeksema et al., 2008; Schacter et al., 2017; Seli et al., 2016). As can be seen in Figure 2a, the further the deep decision tree goes into the future, the higher the number of possible outcomes.

So far, we have focused on action selection in monadic contexts (e.g., deciding which kind of cake to bake), in which pursuing an action does not affect another person. However, in the dyadic context of social interaction, the recursive structure of action selection additionally becomes nested (Fig. 2b). To unroll the nested structure and give a grasp on what it actually means: Agent 1 is engaged to infer the hidden states of the pertaining situation. In a dyadic context, this involves the mental states or beliefs of an interactive partner (Agent 2). Inferring the mental states of another person is commonly termed *mentalizing* or *theory of mind*^1,2^ (Lehmann & Kanske, 2022; Premack & Woodruff, 1978). However, the interactive partner is also inferring the mental state of Agent 1. This leads to another higher level of inference: Now, Agent 1 can also infer the belief of Agent 2 about the mental state of Agent 1. Moreover, Agent 1 can infer how Agent 2 thinks that Agent 1 thinks about the mental state of Agent 2, and so on. All these inferred states are not directly accessible and must be deduced from sensory observations. Thus, they are furnished with high epistemic uncertainty (FeldmanHall & Shenhav, 2019; Wheatley et al., 2019). The described inference processes target only the current state of affairs. However, the exploration of action opportunities that are aimed at counterfactually imagined preferred future states all entail this kind of nested inferences. For example, in a dyadic situation I might be hesitant to look at my watch (out of curiosity about how long a colleague and I have chatted), as I expect that me looking at my watch will lead to my colleague thinking that I intended to signal that I do not have time to chat. Selecting appropriate actions becomes more complex, as there are more paths to compute in parallel (FeldmanHall & Nassar, 2021; FeldmanHall & Shenhav, 2019; Fig. 2b).

As opposed to those second-person situations described above, in experiments on social cognition, mental-state inferences commonly look differently (for an overview, see Schurz et al., 2021). A frequent task is to have participants view a cartoon and let them infer what must be the beliefs of the displayed characters (Wimmer & Perner, 1983). It goes without saying that the recursive depth of belief inference is limited to the first, noninteractive level of participants’ beliefs about the beliefs of a cartoon character; the cartoon character obviously cannot reciprocate. In contrast, second-person experimental contexts capture the idiosyncrasy of social interaction, namely that the belief of the participant is inferred by another (“I think that you think that . . .”; Ramstead et al., 2016). Here, beliefs and inferences evolve from a kind of one-way street, where one person monadically predicts the mental states of another, to reciprocal predictions between interaction partners. By contrast, looking at one’s watch while trying to infer beliefs of cartoon characters would not affect the interpersonal context. Thus, selecting an action in a second-person context inevitably creates *mutual historicity.*^3^ At this level of complexity, people effectively “think through the minds of others,” filtering their own perceptions through what they believe are the beliefs of others^4^ (Ramstead et al., 2016; Veissière et al., 2020).

We argue that the recursive exploration of a deep tree structure, in the service of action selection, is a primary form of information processing in social interaction that gains a genuine social aspect by the nestedness of reciprocal inferences. This kind of deep tree search has recently been proposed as a parsimonious explanation for the processes underlying, at least, some features of default-mode network activity (Dohmatob et al., 2020). More specifically, this information processing can be broken down into two kinds of computations: first, inferences about current and potential hidden states (Fig. 2c) and second, the counterfactually imagined sensory observations, given those hidden states. At a neural level, it has been shown that (hidden) state inference (which entails the evaluation of epistemic affordances) yields activity in the medial prefrontal cortex (Berkay & Jenkins, 2023; Gershman & Uchida, 2019; Starkweather et al., 2018). The sensory consequences of future hidden states are presumably processed in the TPJ as a multisensory prediction of observable outcomes (Dohmatob et al., 2020; Masina et al., 2022). Increased activation in these parts of the default-mode network during actual, second-person interaction (compared with observing others without interacting), in our view, are elicited by an increased depth in deep tree search. Importantly, we suggest that the recursive exploration of a deep tree structure during social interaction is likely—to a large degree—happening in an automatic and implicit mode, which might make social action exploration seem effortless. Contextual or other prior information (e.g., mutual historicity) may provide prior expectations that could additionally narrow down hypothetical alternative states, either through priors on specific states or a more precise likelihood mapping (see **A** in Fig. 2c) between hidden states and sensory outcomes. Cognitive phenomena such as episodic prospection, mind wandering, and mentalizing might draw on this primary form of information processing and thus could elicit activity in the aforementioned components of the default-mode network (Bzdok et al., 2012; Christoff et al., 2016; Schacter et al., 2017; Schurz et al., 2014, 2021; Seli et al., 2016).

Some studies explicitly model recursive interpersonal processes by using dyadic games in a virtual interactive space (Fig. 1d; Coricelli & Nagel, 2009; Devaine et al., 2014; Hampton et al., 2008; Hill et al., 2017). Here, the mPFC and the TPJ were shown to be involved in processing a higher depth of recursion and in accounting for how one’s own actions influence the subsequent actions of an opponent (Coricelli & Nagel, 2009; Hampton et al., 2008). Disrupting the right TPJ led to diminished connectivity between the TPJ and the mPFC, resulting in deficits in estimating how one’s own actions affected the opponent’s behavior (Hill et al., 2017). Interestingly, the engagement of the TPJ and the mPFC—within social interaction—varies with the degree to which an opponent is perceived as “having a mind” (Krach et al., 2008; Takahashi et al., 2014). Opponents to which one ascribes an increased ability to infer mental states (and, thus, to have more recursively nested thoughts) elicit a stronger activation within those regions. Also, when a computer agent is equipped with and changes between different strategies, the increased levels of uncertainty and hidden-state inference elicit greater activity in the mPFC (Yoshida et al., 2010).

### The selection of action

Having explored possible action opportunities, the individual concerned faces a nontrivial question, namely, which of those actions should I select? In the active-inference framework, action is usually considered as a set of beliefs about which action one is undertaking—also referred to as a *policy* (π in Fig. 2c); it models action selection as a kind of self-fulfilling prophecy. As outlined above, social-information processing is inherently furnished with epistemic uncertainty and ambiguity. Now, a deep tree exploration of possible action alternatives and their counterfactually imagined action outcomes (and their respective hidden states) might point to an action that best resolves ambiguity and uncertainty about the state of affairs.

Such a disambiguating policy goes along with a high precision—that is, high confidence in one’s own beliefs about action outcomes (Corcoran et al., 2020; Hesp et al., 2020). This precision signaling is believed to be carried out via dopaminergic transmission in the ventral parts of the basal ganglia (i.e., the ventral striatum; FitzGerald et al., 2015; Friston et al., 2014; Parr & Friston, 2017, 2018; Schwartenbeck et al., 2015). Indeed, this view is substantiated by phylogenetic considerations, according to which a problem as fundamental as action selection must have evolved as early as the appearance of basal ganglia (Cisek & Kalaska, 2010). Dopaminergic signaling may have originally served to arbitrate between local exploitation and long-range exploration (Cisek, 2019; Hills et al., 2015). Interestingly, the precision of beliefs about policies determines how vigorously an action is conducted (Carland et al., 2019; Stephan et al., 2016).

We argue that precision signaling to select actions that have been recursively explored in a deep tree search is a core computation that plays a crucial role in a second-person context. This computation is carried out or signaled in ventral parts of the basal ganglia, which has been consistently shown to be activated in second-person neuroscience studies (for reviews, see Feng et al., 2021; Redcay & Schilbach, 2019). Evidence supports this idea: For example, in a recent study that involved recursive thinking in interpersonal strategic interactions, in which participants had to engage in higher-order belief inferences about another person, both the ventral striatum and the default-mode network showed heightened activity (Zhen & Yu, 2021). Importantly, we do not consider the process of action selection and precision signaling as a purely rational (i.e., context-free) process. Instead, it is tightly linked and informed by the affective states of an interacting agent that mediate a variety of contextual—and possibly hedonic—priors (for a review and formal account, see Hesp et al., 2021).

Besides selecting which action is about to bring the most favorable sensory input, it is crucial to decide when an action ought to be performed. Due to its ever-evolving nature, interactional behavior carries and conveys crucial information on the basis of the timing of an action. This makes both the demand on selecting an action and the corresponding counterfactual belief updates highly dynamic (Schirmer, Meck, & Penney, 2016). For example, naturally occurring, automatic smiles follow a stereotypical temporal pattern (Schmidt et al., 2003). Alterations of this pattern may make a smile seem less spontaneous and more forced. During interpersonal conversations, longer-than-normal gaps in turn-taking signal a semiotic meaning, such as a negative response or trouble in understanding an intended message (Kendrick, 2015; Levinson & Torreira, 2015). Similarly, disgust expressions of longer duration convey a more negative valence (Schirmer, Ng, et al., 2016). You might also think of a situation in which you are in a conversation with two friends, and you want to tell a story (as it matches the current topic of the conversation), but the topic quickly evolves in another direction (as one of those friends tells of something related but with a different twist). As a result of the changing context, the story that you wanted to tell now seems misplaced. The deep tree in Figure 2b can be seen as a snapshot of a particular time that would cover different actions and, thus, look different in the future. Social situations are inherently dynamic and volatile, which adds a layer of uncertainty. Precision signaling to select actions that have been recursively explored in a deep tree search, thus, also facilitates real-time second-person interactions in the dimension of time. Interestingly, within the default-mode network, the mPFC and the TPJ integrate information over longer timescales (over tens of seconds) that are relevant for social inference (Hasson et al., 2015; Hasson & Frith, 2016).

In this section, we have argued that selecting actions requires recursive exploration (and their counterfactual imagined sensory depiction) of action opportunities in components of the default mode network (i.e., the mPFC and TPJ). Importantly, because of the nested structure of the recursive exploration in interactive, second-person contexts, the computational demand on action exploration is of higher complexity, which is reflected in increased activation of those areas. Moreover, we argued that precision weighting to select actions that have been recursively explored in a deep tree search goes along with activity in ventral parts of the basal ganglia. This activity is increased in second-person contexts, as there are higher demands on selecting an action because of higher nested recursion and implicit neural dynamics. A second-person approach exposes the unique processes that play a critical role in the emergence of social interaction. Adopting an active-inference perspective adds foundations about computational principles enabling the interaction in second-person contexts.

## Active Inference and Internal Action

In the previous section, we discussed deep tree exploration of possible action outcomes and the process of choosing among them. We argued that such exploration is the primary computation behind activity in the mPFC, TPJ, and ventral parts of the basal ganglia that have been reported in second-person neuroscience studies. In this section, we consider commonly reported activity in parts of the salience network on the one hand, particularly the anterior cingulate cortex (ACC) and the anterior insula (AI), and parts of the frontoparietal network on the other hand (for reviews, see Feng et al., 2021; Redcay & Schilbach, 2019). In the following, we describe the underlying processes under active inference. We argue that these computations are closely related to internal bodily processes that have been largely neglected in traditional spectator approaches (Engel et al., 2013; Fotopoulou & Tsakiris, 2017; Thompson & Varela, 2001).

### The computation of transition uncertainties

In the active-inference framework, the relation between actions or beliefs about actions (red circles in Fig. 2) and subsequent beliefs about counterfactual observations (blue circles in Fig. 2) is furnished with uncertainty about state transitions (i.e., “How likely is it that a certain state changes from State 1 to State 2 following my action?”; **B** in Fig. 2c). The ACC has consistently been associated with the evaluation of transition probabilities, linking actions to states, and with monitoring whether the observed state transitions matched the preceding action (Akam et al., 2021; Behrens et al., 2007; Dayan & Yu, 2006; Parr & Friston, 2017, 2018; Payzan-LeNestour et al., 2013; Sales et al., 2019). It is thought that this is realized via noradrenergic projections from the locus coeruleus. Accordingly, administering noradrenergic antagonists has been found to impair the processing of transition uncertainty (Marshall et al., 2016). Importantly, transition uncertainty (or volatility) can be represented hierarchically. Beyond merely evaluating volatility at some time, it is also important to know how this kind of uncertainty fluctuates over time and across contexts (Parr & Friston, 2017).

We argue that in second-person neuroscience studies, the observed increase in neural activity in the ACC can be explained by appealing to the higher computational demands on the evaluation of transition probabilities between actions and states. A higher demand might entail more action alternatives to evaluate. These have at least two sources (compared with observational studies): first, the nested recursive structure of deep tree exploration of action, and second, the dynamic ever-evolving fluctuation in the action opportunities.

### The adaptation of the interoceptive bodily milieu

The information that is processed about possible courses of action in the external world certainly influences, and is influenced by, internal bodily cycles (e.g., respiratory, visceral, gastrointestinal; Khalsa et al., 2018; Petzschner et al., 2017). The relation between internal cycles and exteroceptive information—which might also be construed as the first-person subjective frame (Park & Tallon-Baudry, 2014)—is constantly fluctuating, demanding refinement in the budgeting of bodily resources. For example, when you want to initiate a movement (e.g., getting up from a chair), your bodily systems must become attuned to the demands accompanying the movement (e.g., raising blood pressure to ensure a stable blood circulation in an upright position). This alteration of set points to suit the demands of the situation is known as *allostasis* (Corcoran & Hohwy, 2019; Schulkin & Sterling, 2019; Sterling, 2012). It is proposed that interoceptive predictions—originating in granular cells of the AI—function as a set point modulator (Gu et al., 2013) that predicts and determines in which range a bodily system should operate (Livneh et al., 2020). Interestingly, both the AI and the ACC are furnished with large spindle-shaped von Economo neurons (VEN) that are particularly suitable to convey neural information over longer distances (i.e., to the body; Allman et al., 2005, 2011; Hodge et al., 2020).

The attunement of interoceptive systems to the demands of interacting with the world is evident in multiple internal cycles, such as the respiratory tract (Park et al., 2020), the digestive system (Levinthal & Strick, 2020), or the visceral system (Allen et al., 2022). Importantly, this attunement is bidirectional, suggesting a kind of circular causality. For example, voluntary movements are more likely conducted during exhalation (Park et al., 2020), while preparing for a movement is accompanied by cardiac slowing (J. I. Lacey & Lacey, 1970). On the other hand, exteroceptive information that requires higher levels of attention leads to a deceleration of the heart rate, probably downregulating internal noise in order to increase precision of the external information (B. C. Lacey & Lacey, 1978; Sokolov, 1963).

During social interaction, the attunement of internal bodily systems to the environmental demands is crucially caused by demands of the exchange between two agents. For example, during a conversation between you and your interlocutor, you might adjust your body (by inhaling) in anticipation of a state transition, which is taking your turn to say something (Rochet-Capellan & Fuchs, 2014). Although it is possible to attune to another person via third-party observation (e.g., when watching somebody who is telling a sad autobiographical story; Kanske et al., 2015, 2016), real interpersonal (second-person) situations generate a stronger drive to do so, making the relationship between two social agents more vivid. Accordingly, during social interaction, multiple internal systems attune to internal systems of the interactive partner, resulting in synchrony (Hoehl et al., 2020; Koole & Tschacher, 2016; Kupper et al., 2015; Mayo, 2020; Tschacher et al., 2017). For example, interacting dyads show more stable and shared heart-rate dynamics than noninteracting dyads (Fusaroli et al., 2016).

Taken together, we argue that the ACC and AI (as parts of the salience network), act in concert to (a) compute transition probabilities and track the matching between actions and states, and (b) attune internal systems to environmental demands as a result of fluctuating states. This aligns well with literature that views the salience network as a hub for behavioral adaptation in light of changes in environmental contingencies (Poe et al., 2020; Uddin, 2015).

### The adaptation of the proprioceptive bodily milieu

Beside interoception, important bodily functions with regard to second-person interaction are captured in the proprioceptive domain, such as the sensorimotor communication (Pezzulo et al., 2019). Sensorimotor communication is the aspect of communication that is visible to the interactive partner and involves parts of the body (muscular, tendon, and articular) that are oriented “toward the world” (Fuchs, 2020, p. 3). As we have discussed, in active inference, the motor cortex is not viewed as an area that issues motor commands; rather, it is involved in issuing predictions (Adams et al., 2013; Hipólito et al., 2021). Specifically, somatomotor predictions about sensory consequences of an action are sent to the spinal cord, where they are compared with ascending (primary) proprioceptive afferents. The comparison of both ascending and descending information results in a prediction error that can be resolved by action (i.e., classical motor reflexes in the spirit of the *equilibrium-point* hypothesis; A. G. Feldman, 2009)—fulfilling the issued prediction of the motor cortex. Similar to ACC and AI, the motor cortex harbors large spindle-shaped neurons (Betz cells; Rivara et al., 2003) that are also suitable to convey information over long ranges. Additionally, the somatosensory cortex receives information about the sensory consequences of an action, which enables more refined predictions for future actions.

The recursively nested and dynamically changing nature of social interaction influences the prediction of our bodily actions toward the world, that is, toward interactive partners. Because interactive partners have a life on their own, which in turn has an influence on us, our sensorimotor predictions need to account for a higher level of contextual variability. Accordingly, higher levels of dynamicity in motor production go along with an increase of activity in somatomotor and somatosensory cortices (Neely et al., 2013). Such results can also be observed in sensorimotor interaction within a second-person neuroscience approach (e.g., Chauvigné et al., 2018; Kokal et al., 2009). For example, Kokal et al. (2009) engaged participants in a joint action task, in which the participant and another person were required to arrange two sticks in a previously indicated assembly. Here, somatomotor and somatosensory cortices showed superadditive activation for joint actions compared with its constituents (observing action and executing action alone), suggesting a higher complexity due to bidirectionality. Similarly, performing improvised joint hand movements with another person also activated somatomotor and somatosensory cortices (Chauvigné et al., 2018).

In this section, we have argued that the nested recursive structure of deep tree exploration of action (drawn from the active-inference framework) and the dynamic, ever-evolving fluctuation in the action opportunities within second-person contexts entails two core things. First, it leads to a higher computational demand on the evaluation and monitoring of transition probabilities, which elicits higher ACC activity. Second, it results in more environmental variability and fluctuating states, which calls for an increased attunement of internal (via the AI) and proprioceptive (via parts of the frontoparietal network) systems. Here, a second-person approach exposes (interoceptive and proprioceptive) bodily processes that enable and transmit social interaction. Adopting an active-inference perspective adds the underpinning computational principles in this bodily social interaction.

## Integrating the Mechanisms of Second-Person Neuroscience

In the previous sections, we attempted to delineate delimited processes underlying social interaction within a second-person context. We argued that moving from the detached, spectator position (third person) to an interactive, second-person perspective introduces nested, recursive exploration of action opportunities that mainly draws on counterfactual simulation of states and their respective sensory outcomes in the mPFC and the TPJ—which we can model using the tools of sophisticated and sociocultural active inference. Furthermore, the implicit multitude of action opportunities leads to a higher demand on selecting a course of action, which engages ventral parts of the basal ganglia. As a result, there emerges a higher demand on Bayesian belief updating in the ACC, which leads to a more flexible attunement of interoceptive bodily systems, conveyed via the AI. Similarly, somatomotor and somatosensory cortices are engaged in attuning the proprioceptive bodily system, in order to act toward the interactive partner.

However, importantly, these processes should by no means be considered as unfolding in an isolated manner. Rather, it is their complex interplay that allows us to master the hurdles of interaction and lets us navigate the social environment (more or less) smoothly. A few studies investigated the interplay of these processes and delivered valuable insights about their mutual influence. Hesp et al. (2020) examined how the policy selection of an artificial agent is influenced by recursively sampled action outcomes. Interestingly, the precision or the confidence about one’s own actions is reduced every time a negative outcome is imagined. The higher the engagement in counterfactual iterations, the more often negative outcomes are encountered mentally, leading to a substantive reduction in confidence—that is, overthinking, rumination, and catastrophizing. Tragically, perhaps, this reduction of confidence affects future actions: Imagined outcomes become negatively biased, resulting in a vicious circle, effectively leading to more caution in action selection.

Interestingly, altered confidence about one’s own action models might also affect the degree to which an environment is perceived as being volatile. For example, in a simple reversal-learning task in which contingencies between stimuli and subsequent outcomes were manipulated to be more or less volatile, Weiss et al. (2021) let participants either interact with, or merely observe, the presented evidence. Having the opportunity to act, reliably led to impressions that the environment was stable and less volatile. The relation between volatility and action selection can also be looked at from the other direction. For example, some have argued that volatility expectations affect policy selection—that is, higher environmental volatility leads to adopting simpler policies (i.e., habits) that bring higher precision on action outcomes in a more uncertain world (FitzGerald et al., 2014; Fradkin et al., 2020). Along these lines, Hesp et al. (2021) have proposed that action selection becomes dominated by prior policy preferences (i.e., habits) when confidence in one’s action model becomes too low.

The aforementioned interlocking mechanisms play a role in real social interaction; for example, counterfactually overthinking potential interpersonal actions and their consequences might diminish one’s confidence in interpersonal actions, leading to more passive or apathetic behavior (Hezemans et al., 2020). This, in turn, would lead to experiencing the social situation as more volatile, which in turn promotes the propensity to enact behaviors whose action outcomes are highly predictable.^5^ Predictable action outcomes in social situations can be attained by making oneself more predictable to the interactive partner and thereby diminishing mutual prediction error (i.e., prediction error received from the interactive partner; Theriault et al., 2021).

At a neural level, we can note that truly interactive interpersonal situations (Figs. 1d and 1e) foster the recruitment of all of the brain networks discussed above (Cavallo et al., 2015; Chauvigné et al., 2018; Rauchbauer et al., 2019; Redcay et al., 2010; Xie et al., 2020). This might give credence to the notion of a complex interplay between the mechanisms relevant to social interaction (nested recursion in mutual and reciprocal mental-state inferences, inter- and intrapersonal bodily attunement, and policy selection). Importantly, in most cases, the brain activity that enables social interaction is contrasted with activity derived from either doing a task alone or from observing another without interacting. This means that the brain shows more widespread activation when being involved in a social interaction. This finding nicely fits simulation-based research showing that minimal agents exhibited a higher degree of neural complexity when interacting with other agents compared with interacting with “ghosts” displaying behavior that was not influenced by their environment (Candadai et al., 2019; Reséndiz-Benhumea et al., 2020, 2021). Interestingly, the level of neural complexity that one observes while interacting with other agents exceeded levels that could be generated by an isolated agent, suggesting that single agents become parts of a dynamically extended system (Froese et al., 2013). The increased brain activity during social interaction might indicate such a transition from an isolated brain toward a whole brain-body-environment-body-brain system (cf. Lewis et al., 2011, 2017; Powell et al., 2010).

## Concluding Remarks and Future Directions

In this article, we have considered the neural mechanisms of second-person social interaction through the lens of active inference. The mechanisms that we have discussed could be framed as computational operationalizations of the core intra- and interpersonal phenomena that intertwine and enable actual social interactions. We outlined the neural mechanisms that play a role when we move beyond observing and are actually engaged in real-time (second-person) social interaction. We have argued that being embedded in an interpersonal context introduces the interesting phenomenon of nested recursive exploration of action opportunities that mainly draws on counterfactual simulation of outcomes under various hidden states in parts of the default-mode network. Furthermore, the multitude of action opportunities that are ever evolving and fluctuating leads to a higher demand on selecting a course of action, which is processed in ventral parts of the basal ganglia. As a result, there emerges a higher demand on computing transition uncertainties in the ACC, which leads to a more flexible attunement of interoceptive bodily systems (conveyed via the AI). Similarly, somatomotor and somatosensory cortices are engaged in attuning the proprioceptive bodily system in order to act toward the interactive partner.

Importantly, these processes are entailed when going beyond the mere observation of social stimuli and toward an actual engagement in real-time reciprocal interaction. As such, the above-mentioned processes are here thought of as crucial for the emergence of genuine social interaction and vice versa. More concretely, we claim that such processes rely on domain-general uncertainty-minimizing belief updating, which, in turn, has been shaped in—and through—social interaction across various scales, ranging from phylogenesis and culture to ontogenesis and everyday learning and psychophysiology (Bolis & Schilbach, 2020a). Consequently, it is not always easy, or even possible, to make a clear distinction regarding what might be uniquely social (Bolis et al., 2022; De Jaegher et al., 2010). What appears strictly private at times (e.g., self-regulation) might have been formed interactively (interpersonal regulation; Kiel & Kalomiris, 2015) and thereby presupposes the whole historicity of social interactions. Various aspects—ranging from conscious decision-making all the way down to interoception—might all be thought of as interactively formed along development and beyond (Bolis & Schilbach, 2020a; De Jaegher et al., 2010; Fotopoulou & Tsakiris, 2017). One component that arguably constitutes a uniquely social aspect is *nested recursion*, which necessarily requires at least two individuals. In our view, nested recursive inferences yield a specific profile of uncertainty that mainly involves the other processes discussed (higher demand on policy selection, higher transition uncertainty, and the demand to attune various bodily systems). High (epistemic) uncertainty is presumably an aspect in which active social engagement converges with passive and observational forms of social processes. We emphasize that active engagement goes along with a disproportionately higher degree of uncertainty that, however, can be resolved via action.

In order to apply insights gained from laboratory experiments to full-blown social interaction, researchers need to adopt a second-person neuroscience methodology. Phenomena that are unique to true social interaction—such as the ones operationalized via nested recursion in mental-state inferences, transition uncertainty, and high demand on action/policy selection—need not only be preserved but explicitly targeted in experimental paradigms. Considering these interlocked mechanisms all at once, in nonrestricted and free social interaction, might be computationally less tractable, but the complexity of certain parameters could be diminished. For example, paradigms could limit and manipulate action possibilities of interacting agents and thereby also get a grasp on the level of interpersonal volatility. Importantly, neuroimaging data should be complemented by various bodily measures, such as heart rate, skin-conductance response, respiratory rhythm, and pupil dilation (among others), but also by psychological states (cf. two-person and collective psychophysiology; Bolis et al., 2022; Bolis & Schilbach, 2018). Processing these physiological measures from two interacting persons might be carried out using methods from dynamical-systems theory. Here, for instance, altering states of synchrony and asynchrony might be cast as state transitions, targeting the volatility parameter. Interdisciplinary efforts of psychologists, neuroscientists, and engineers or computer scientists could fabricate testable behavioral as well as neurophysiological hypotheses derived from generative computational models, harnessing advancements from the active-inference framework.

Concrete research questions, for instance, could concern the level of nested recursion. Does a higher need for nested hidden-state inferences (i.e., nested recursion) lead to a higher level of assigning precision to a certain policy (accompanied by higher levels of dopaminergic signaling)? Is this relation monotonic, or does policy precision start to decrease at a certain level of overthinking? How are these relations altered with increasing or decreasing levels of action opportunities? A strong candidate for parameter manipulation would also be the transition uncertainty (i.e., how likely is it that a particular action leads to a particular state transition). Does an increasing level of transition uncertainty (e.g., due to more erratic behavior of an interactive partner) lead to more nested hidden-state inferences (thereby accumulating expected evidence for potentially disambiguating policies) or rather to habitual behaviors (i.e., actions that have elicited preferred outcomes in the past)? Potentially, this relation would also be characterized by a nonlinear function. Where is a potential and individualized optimum between balancing nested hidden-state inferences with more habitual policies? Here, the impact of manipulated transition uncertainty on bodily variables might be explored. Does erratic behavior of an interaction partner (i.e., high transition uncertainty) lead to a reduced heart-rate variability? Also, pharmacological studies could be beneficial for the investigation of a second-person active-inference model. For example, dopamine agonists (e.g., dopamine reuptake inhibitors) could lead to assigning higher precision in social policies, whereas noradrenaline agonists might aid learning about the contextual variability of another person’s states. Another interesting implementation could concern the mutual influences of individual parameters. For instance, how does the precision about policies in one person affect the same parameter in an interactive partner (Friston & Frith, 2015; Isomura et al., 2019)?

A concrete implementation on a higher and more collective level could be pursued in the form of a meta-Bayesian framework, situated in dynamical-systems theory (cf. Bolis & Schilbach, 2017; Brandi et al., 2019). Such a multiscale framework will aim to track cognition and action via distinct active-inference models, which will be targeting each individual in a given interaction while still potentially being fused on a collective (meta-Bayesian modeling) level of behavior. Moving the focus from the observation of individual passive observers toward a multiscale tracking of dyads and groups of interactors could even allow people to assess “whether and how interpersonal coordination in real-time social interactions might actually serve as a prior and modulate the need for inferences about hidden causes of social behavior” (Bolis et al., 2017). Such an interpersonal active-inference approach might provide a formal understanding of not only individual-specific but also dyadic and group-level dynamics. These completely new avenues of research have the potential to revolutionize our understanding of social interaction at various layers and scales, ranging from the individual to the collective.

## Transparency

*Action Editor:* Leonel Garcia-Marques

*Editor:* Interim Editorial Panel

## Notes

1. Addressing critique on the concept of theory of mind, a recent theory has referred to this cognitive process as “thinking through other minds” (TTOM; Veissière et al., 2020).

2. Sometimes mentalizing or theory of mind is broken down into implicit (spontaneous and unconscious) and explicit (controlled and reflective) processes (Evans, 2008). Our considerations apply to both modes; however, we note that during real-time social interaction implicit forms of mentalizing are predominant.

3. Mutual historicity emerges from action selection within a social context. As soon as a person engages in a dyadic interaction, the interactive partner engages in inferences about the person’s underlying mental states. Those inferences will shape future (person-specific) prior expectations and the precision of the likelihood mapping between observations and hidden states (A in Fig. 2c), narrowing down a variety of hypothetical states. Additionally, one’s own actions will be selected on the basis of inferences about the other person’s states and state transitions (B in Fig. 2c). Every behavior influences certain parameters in this model and will be shaped on the basis of mutual historicity.

4. There are many examples of how an interactive dyadic context and its accompanying nested recursive thought patterns alter behavior compared with a noninteractive context. For instance, in a joint-attention paradigm, nested recursive thoughts (such as “I [don’t] want to let the other guy down” or “I [feel] that Alan [is] better at the task than I [am]”; Caruana et al., 2017, p. 13) lead to faster reaction times when responding to—and initiating—gaze shifts (Caruana et al., 2017). In another study, Krishnan-Barman and Hamilton (2019) showed that movement patterns were more likely to be imitated by a follower when the leader could watch the follower’s action—a phenomenon that was, it was argued, due to a desire to affiliate. Similarly, kinematic patterns have been shown to be distinct when one is acting in isolation compared with the same action in a social context (Becchio et al., 2010).

5. Interestingly, the experience of volatility within the social environment and the subsequent preference for risk-averse behavior have been argued to play an important role in the emergence of depressive symptoms (but see Badcock et al., 2017, for a comprehensive review). An example of this risk-averse response to volatility might be withdrawing from the volatile environment and consequently experiencing more predictable outcomes.
